# Recent advances in melt electro writing for tissue engineering for 3D printing of microporous scaffolds for tissue engineering

**DOI:** 10.3389/fbioe.2022.896719

**Published:** 2022-08-17

**Authors:** Sebastian Loewner, Sebastian Heene, Timo Baroth, Henrik Heymann, Fabian Cholewa, Holger Blume, Cornelia Blume

**Affiliations:** ^1^ Institute of Technical Chemistry, Leibniz University Hannover, Hannover, Germany; ^2^ Institute of Microelectronic Systems, Leibniz University Hannover, Hannover, Germany

**Keywords:** 3D printing, melt electro writing, scaffolds, tissue engineering, electrospinning

## Abstract

Melt electro writing (MEW) is a high-resolution 3D printing technique that combines elements of electro-hydrodynamic fiber attraction and melts extrusion. The ability to precisely deposit micro- to nanometer strands of biocompatible polymers in a layer-by-layer fashion makes MEW a promising scaffold fabrication method for all kinds of tissue engineering applications. This review describes possibilities to optimize multi-parametric MEW processes for precise fiber deposition over multiple layers and prevent printing defects. Printing protocols for nonlinear scaffolds structures, concrete MEW scaffold pore geometries and printable biocompatible materials for MEW are introduced. The review discusses approaches to combining MEW with other fabrication techniques with the purpose to generate advanced scaffolds structures. The outlined MEW printer modifications enable customizable collector shapes or sacrificial materials for non-planar fiber deposition and nozzle adjustments allow redesigned fiber properties for specific applications. Altogether, MEW opens a new chapter of scaffold design by 3D printing.

## Introduction

Tissue engineering (TE) is an innovative interdisciplinary research topic with the purpose to produce sustainable substitutes for damaged tissue. For each artificially produced tissue, different prerequisites have to be fulfilled, depending on the detailed histological build of that particular tissue. Therefore, TE needs to integrate different fields such as material science, cell biology and a technically advanced bioreactor design in order to achieve a successful and physiologically rational tissue build-up. Successful TE-products are supposed to repair and regrow damaged tissue, often times implantable matrix constructs, so called scaffold structures, are needed to guide cell ingrowth, differentiation and tissue formation as well as an optimal adaptation in a host tissue milieu.

While there are many scaffold fabrication techniques like emulsion freeze drying (Sultana and Wang, 2008; Qian and Zhang, 2011; Sultana and Wang, 2012), gas foaming (Manavitehrani et al., 2019; Luo et al., 2021; Chen Y et al., 2021), porogen leaching (Bhaskar et al., 2018; Poddar et al., 2021; Santos-Rosales et al., 2021) or phase separation (Ferrolino et al., 2018; Abzan et al., 2019; Zeinali et al., 2021), more recently additive manufacturing (AM) gained an increased interest for scaffold fabrication (Shick et al., 2019; Szymczyk-Ziółkowska et al., 2020; Veeman et al., 2021). Generally, the term AM refers to a group of additive manufacturing techniques used to produce a scale model of a physical part or assembly as a three-dimensional model as quickly and inexpensively as possible. Rapid prototyping is one of the most mature applications for additive manufacturing technologies. In an industrial context, 3D printing is usually referred to as additive manufacturing; in this review, we speak of 3D printing as a specific sub-feature of AM. In AM three-dimensional structures are build up in a layer-by-layer manner by depositing material using digital data. In contrast to other manufacturing techniques, AM allows the superior control and reproducibility of scaffolds by using computer-aided design and control. AM and 3D printing techniques are particularly interesting for TE due to the possibility of using information from imaging techniques for the creation of individual implants (Javaid and Haleem, 2018; Javaid and Haleem, 2020).

Electrohydrodynamic atomization techniques gained increased interest in scaffold production for their ability to form micro- to nanoscale structures. These rely on interactions between electrostatic energy and working fluids, and are powerful in nanofabrication, modification, and writing. They include electrospinning, electrospraying, e-jet printing and among them, melt electrospinning techniques are drawing increasing attention (Liu et al., 2022; Yu et al., 2022). Melt electrospinning, a technique in which micro-fibers are fabricated using a polymer melt without the need for a solvent, was first described in publications in the eighties of the last century (Larrondo and St. John Manley, 1981a; Larrondo and St. John Manley, 1981b; Larrondo and St. John Manley, 1981c). Lately, the potential to adapt melt electrospinning to additive manufacturing methods was established termed melt electro writing (MEW). MEW is an additive manufacturing method that was first introduced in 2011 and combines principles of electrospinning and melt extrusion based methods (Brown et al., 2011). Only recently, MEW also gained increased interest for TE to produce precise and defined microfibers and to build up structures with a tunable microarchitecture and external shape.

**FIGURE 1:**
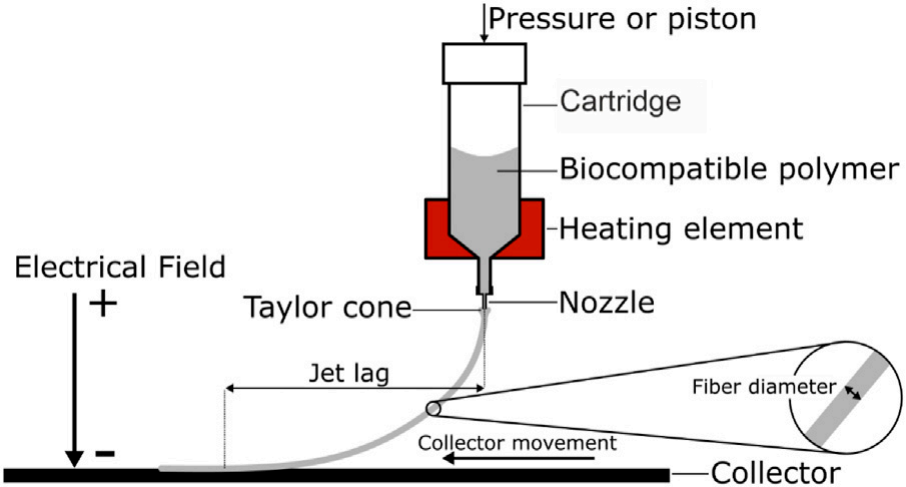
Schematic melt electro writing process: A pneumatic or volumetric feed is used to extrude a heated polymer through a nozzle. Due to the electrical field, the polymer droplet at the nozzle tip forms a conical shape, the Taylor cone. From the Taylor cone a microscale fiber is drawn towards the collector. Depending on the material feed and the collector speed, the result is a curved fiber path and an offset between fiber release and deposition, the jet lag.

MEW uses materials that are flowable by melting, termed thermoplastic materials, and is therefore especially suitable for processing biocompatible polymers. The general principle of MEW can be divided in two steps (Figure 1): first the molten material is extruded through a nozzle using air pressure or volumetric dispensing (e.g. piston). Secondly, a small droplet is created at the end of the nozzle. Similar to solution electro spinning, a high voltage (HV) is applied between the nozzle and the print bed. The HV exerts an electrostatic attraction on the molten material, and a so-called Taylor cone forms at the end of the nozzle. The Taylor cone is the cone-shaped deformation of a liquid surface exposed to an electric field. This deformation is the result of a balance of forces between gravity, surface tension, internal hydrostatic pressure, external gas pressure and the electric force resulting from the applied electric field. In the case of MEW, a microscale polymer filament is created at the end of the Taylor cone, which develops into a fiber and is drawn towards the collector. The applied voltage further stabilizes the polymer jet and prevents Plateau-Rayleigh instabilities, which would cause the jet splitting into droplets (Stachewicz et al., 2017). The electrical field accomplishes a steady material flow and plays a major role in MEW by regulating important properties such as flight path and fiber diameter of the fiber jet. Various configurations exist were either the nozzle is charged and the print bed is grounded, or vice versa a grounded nozzle is used and a print bed is charged. Either way, a net force is established between nozzle and collector to attract the jet to the collector.

Other important parameters of the MEW process are the temperature of the polymer melt, the pressure or feed rate, the speed of moving elements such as the collector and/or of the print head and the working distance, defined as the distance between nozzle and collector. By adjusting these parameters, the polymer melt can steadily be extruded and directly written onto a collector. With a coordinated movement of the computer controlled print head and also of the collector, predefined patterns and structures can be produced. 3D-constructs with customizable shape and microarchitecture are obtained by repetitive layer-by-layer fiber stacking. Due to the distance between nozzle and print bed, MEW is a contactless 3D printing technique, contrary to other methods such as fused deposition modeling (FDM).

While Solution electro spinning (SES) is not considered an AM technique, it is extensively used for its ability to form high surface area-to-volume ratio micro- and nanofibers for drug delivery, tissue engineering and filtration. In SES, a polymer solution is pumped through a nozzle (also termed needle or spinneret). Between the nozzle and a collector, a high voltage is applied. The electrostatic field causes a repulsion of the polymer solution at the tip and forms a droplet of conical shape, the Taylor cone. When the repulsing forces overcome the surface tension of the polymer solution, a polymer jet is ejected towards the collector. As the solvent evaporates, the jet experiences perturbations, often called “Whipping”, which causes a jet thinning and a randomly nonwoven deposition on the collector (Kakoria and Sinha-Ray, 2018). MEW has multiple advantages over SES. By using a polymer melt with a high viscosity and a low conductivity, the exact positioning of the fiber is greatly improved and allows accurate stacking of fibers. Also electrical instabilities such as charge repulsion are suppressed due to an increased viscosity. Since no solvent is used in MEW, there is no need for a larger airborne period to allow solvent evaporation, and the distance between collector and nozzle can be reduced considerably. In addition, there is no need to remove volatile solvents and prove the absence of any harmful residual solvent in the MEW-produced TE construct. Rather than via solvent evaporation, the fiber in MEW solidifies by cooling; this happens when or prior to the molten jet touching the collector.

Near-field electrospinning is another techniques used for the fabrication of nano-to micrometer scale fibers for electronics, gas sensors or tissue engineering scaffolds. Similar to traditional (or far-field) SES, a fine polymer fiber is created by polymer solvent blend accelerated towards a charged collector. In contrast to SES, near-field electrospinning achieves controllable fiber deposition by reducing the collector distance significantly to eliminate whipping. In comparison with near-field electrospinning, MEW has the considerable advantage of less complicated and more precise fiber stacking into multiple layers (Nazemi et al., 2022). Only the incorporation of additives or the cumbersome combination with other 3D printing methods enables near-field electrospinning the preparation of high aspect ratio constructs (Kim et al., 2008; Gill et al., 2020). Near-field electrospinning and SES have the general advantage of smaller fiber generation and broader material suitability then MEW.

MEW is capable to produce fiber diameters that are substantially smaller than those generated within other 3D printing techniques, such as FDM. Typically, MEW fibers are 5–40 µm in diameter but can be as small as 800 nm (Hochleitner et al., 2015). In comparison, SES achieves the production of smaller fiber diameters than MEW, but MEW has the potential to exactly determine the deposition of the micro-scale fibers and it allows precise layer-by-layer fabrication of millimeter sized constructs. The latter fact makes MEW such a promising technique for production of precisely structured microporous scaffolds for TE.

This review aims to outline recent advances in control and optimization of MEW with regard to TE applications. Especially the advances of an improved printing resolution and precise deposition over several layers are described. Furthermore, techniques to enable application specific microstructures and methods to broaden the possibilities of MEW such as dynamic fiber diameter, out-of-plane printing, and microscale shifting are outlined.

## Optimization of melt electro writing

MEW is an inherently multi-parametric AM technique and most of the time a trial-and-error-approach is used to determine suitable operating parameters. To accomplish highly precise fiber deposition and desired fiber diameters without defects, we suggest, that several aspects can be taken into account from the outset with regard to a predictable 3D printing result.

To reach stable printing conditions in MEW, an equilibrium between mass flow to the nozzle tip and electrostatic forces pulling material from the forming Taylor cone is needed. A mismatch leads to material aggregates within the Taylor cone until excess material is pulled towards the collector in an oscillating fashion. This phenomenon is often termed “fiber pulsing”, which leads to inhomogeneous fiber diameters. Hochleitner et al. characterized three types of fiber pulsing, phenomena that occur depending on the mismatch. Temporary pulsing is induced by changes in instrumental parameters (e.g. feeding pressure) during the process but also at the time of starting the process. The initial disequilibrium affects the mass flow and causes an oscillating jet profile, until a stable condition is reached. An excess of material flow to the nozzle leads to continuous pulsing, a condition with ongoing change in fiber diameter. A stronger electrical field is needed to stabilize the jet and to induce an equilibrium of mass flow. On the other hand, long bead defects can occur when either a very low voltage or a high pressure is set. Outliners with a multiple of the usual small fiber diameter up to the shape of droplets are deposited in a recurring manner (Hochleitner et al., 2016).

Precise fiber deposition is needed to achieve printing results equal or even superior to other 3D printing techniques such as FDM. Because MEW is a contactless manufacturing process, the process parameters have a decisive role in affecting the fiber deposition. A collector speed above the material deposition rate is necessary to achieve fiber deposition close or equal to the toolpath. This minimum speed is termed “critical translation speed” or in short CTS. Printing speeds under the CTS produce a wave- or loop-like fiber deposition and thus a deviation between toolpath and deposited fiber. Printing speeds above the CTS lead to a jet lag of the fiber with a catenary shaped fiber flight path and a visible angle of the generated fiber. The fiber flight path affects the fiber diameter due to a mechanical stretching effect that thins the fiber while it is attracted to the collector. At the same time, the jet lag when using printing speeds above the CTS, causes a greater deviation between print path and print result with nonlinear print head movements.

The fiber diameter in MEW is dependent on the collector speed, pressure and, to a lesser extent, collector distance while the applied voltage has no significant effect (Dayan et al., 2018). To adjust the fiber diameter, Hrynevich et al. introduced formulas to predict the fiber diameter depending on the change of pressure or on the collector speed with printing speeds above the CTS:

$${\text{D}}_{2}={\text{D}}_{1\;}\times \sqrt{\frac{{\text{V}}_{1}}{{\text{V}}_{2}}}$$ (1)

$${\text{D}}_{2}={\text{D}}_{1\;}\times \sqrt{\frac{{\text{P}}_{2}}{{\text{P}}_{1}}}$$ (2)

The change in fiber diameter can be calculated for a stable process with known values for the fiber diameter (D_1_), for the collector speed (V_1_) and for the printing pressure (P_1_). By inserting the changed pressure P_2_ and collector speed V_2_, the new fiber diameter D_2_ can be estimated. Also parameters for target fiber diameter can be extracted using this formula (Hrynevich et al., 2018).

Besides the pressure and collector speed, the nozzle size is important in order to achieve the smallest possible fiber diameter. Hochleitner et al. achieved replicable submicrometer filaments with 817 ± 165 nm diameter in addition to a high collector speed due to a small nozzle diameter of 0.108 mm (33G) (Hochleitner et al., 2015).

In MEW, the fiber diameter can even be modified during the printing process which is in contrast to other 3D printing techniques. A change in material flow or tool speed can be used to induce fiber flight or material flow changes resulting in a modified fiber diameter. While a decrease in pressure or volumetric material flow induces a smaller fiber diameter, a stabilization period is needed to overcome the temporary oscillating period as pointed out before. In addition, the variation of collector or print head speed or both can be used to increase or decrease the mechanical stretching on the deposited fiber. While an increase in tool speed enhances the mechanical stretching effect and decreases the fiber diameter, it also increases the jet-lag, the deviation of tool position and the fiber contact to the collector. When changing the tool speed in the MEW process, an accelerating or slowdown path is needed until the desired fiber diameter is achieved. Therefore changing the fiber diameter in the printing process has to be planned appropriately to achieve the desired scaffold properties and unneeded parts have to be removed after the print run (Hrynevich et al., 2018). Jin et al. investigated the adjustment of the strand diameter by the adaptation of the speed during the MEW process and developed a strategy to adjust the toolpath depending on the jet lag in a specific scope to achieve accurate strand deposition. By measuring the jet lag as a function of the collector speed and the resulting strand diameter, a reverse speed planning process was established to avoid the error introduced by the jet lag and to generate the desired fiber diameter at the correct location (Jin et al., 2020).

Layer-by-layer deposition is relevant to achieve scaffolds with a tissue specific size. Maintaining precise fiber deposition during increased layer fabrication is to obtain the purposed clinically relevant support structures. One aspect affecting the fiber deposition is the distance between nozzle and scaffold. With each layer, the distance between the nozzle and scaffold decreases, which affects the fiber deposition in subsequent layers due to an altered fiber flight path. To counteract this effect, increments in collector distance have to be used to accommodate the decreasing distance between nozzle and the top layer of printed object (Wunner et al., 2018b; Mieszczanek et al., 2021; Zheng et al., 2021). The increase in working distance decreases the electrostatic forces on the Taylor cone and can therefore lead to material build up which may affect fiber deposition and may cause fiber pulsing. Due to the fabrication properties of MEW, the electrical field also plays a critical role in forming the micro fiber and its precise deposition on to the collector. Therefore, strategies to maintain the electrical field strength are needed to enable a defect-free fiber deposition in a layer-by-layer fashion. Voltage and collector spacing are increased to maintain a constant electric field and adjust the fiber trajectory during continuous layer-by-layer deposition (Saidy et al., 2019; Wunner et al., 2019; Mieszczanek et al., 2021; Zheng et al., 2021). In many cases, the deposited fibers themselves are charged after deposition, influencing the electrical field and interfering in the accurate deposition of subsequent fibers. By this circumstance, the flightpath of the deposited fiber can be severely altered. The increased voltage is needed to compensate the charge of the already deposited fibers and the increased distance to the collector. By adjustment of the electrical field and distance to the printed object, constructs with multiple hundred layers and up to 9 mm in height were successfully produced (Mieszczanek et al., 2021; Zheng et al., 2021).

### Impact of scaffold geometries

While cell responses to different environments are different depending on the cell type, cells show characteristic responses to their surroundings. Thus, an optimal extracellular matrix has to be re-created by 3D printing to optimize culture results and mimic native tissue conditions by favoring optimal cell ingrowth, cell attachment as well as continuous proliferation and migration processes as well as cell alignment and differentiation. Therefore, careful consideration must be given to scaffold architecture, arrangement, and structure size for successful artificial tissue growth (Eichholz and Hoey, 2018; Abbasi et al., 2019; Tourlomousis et al., 2019; Xie et al., 2019). Here, MEW can produce fibers with a diameter range between 0.8–40 µm (Hochleitner et al., 2015) which resemble the size of natural ECM fibers. The most common and relevant ECM components are for example collagen fibrils or reticular fibers (0,2-1 µm Ø), collagen fibers (1–20 µm Ø), elastin fibers (0.1–0.2 µm Ø) (Ushiki, 1992) or fibronectin fibers (5–20 nm Ø) (Chen et al., 1997; Früh et al., 2015).

The fiber diameter also affects the cell migration speed and the migration distance. In addition, an increased cell elongation is observed on larger fiber diameters. When multiple fiber diameters are present, cells generally align orthogonal to smaller fibers (3 µm Ø) and adhere to the surface of thicker fibers (22 µm Ø). When only one fiber diameter is present, cells generally grow randomly (Jenkins and Little, 2019; Xie et al., 2019).

A higher fiber density in parallel to a lower porosity is associated with a higher cell proliferation. Due to the fabrication freedom of MEW, cell proliferation on scaffolds could be regulated by adapting the pore size on specific areas on the scaffold (Xie et al., 2019). Fiber density also affects the cell morphology. Thus denser scaffolds with smaller pores lead to a higher cell roundness, because cells extend across multiple fibers, and in parallel to a lower migration speed. On the contrary, when the fiber distance is increased, cells adhere to single fibers and show an increased alignment in fiber direction, and an increased alignment of the actin cytoskeleton along the fibers is observed. In parallel, an increased migration speed is observed (Jenkins and Little, 2019).

Fiber alignment effects cell morphology and can promote cell differentiation. For example a more spindle shaped elongated phenotype is observed in parallel to an increased fiber alignment, while randomly oriented fibers induce a more rounded cell morphology. Summarizing, the exact determination of fiber deposition and alignment in MEW has an high impact on cell forms and differentiation (Jenkins and Little, 2019).

## Generating scaffold patterns with MEW

MEW scaffolds can be used for a broad range of artificially engineered tissue types, as well for hard tissues such as ligaments and tendons (Hochleitner et al., 2018b), bone (Abbasi et al., 2020; Meng et al., 2021; Wang et al., 2021), cartilage (Peiffer et al., 2020) and for soft tissues such as heart valves, skin (Hewitt et al., 2019), blood vessels (Jungst et al., 2019; Saidy et al., 2020), periodontal tissue (Daghrery et al., 2021) and nerves (Wang Y et al., 2020; Chen Y et al., 2021). This makes MEW a fascinating technology in 3D printing with high potential. An overview of these different tissue scaffolds is provided in Table 1.

**TABLE 1:** Tissue specific scaffolds fabricated *via* MEW.

Tissue	Scaffold description	Citation
Myocardial Tissue	Hexagonal PCL microstructure	Castilho et al. (2018)
	Rectangular or square pore (poly (hydroxymethylglycolide-co-ε-caprolactone) scaffolds	Castilho et al. (2017)
Heart valve	Serpentine PCL architecture to mimic collagen fibers	Saidy et al. (2019)
Ligament and Tendons	Sinusoidal patterns for aligned, crimped collagen fibrils imitation	Hochleitner et al. (2018a)
Skin	PCL blend with bioactive milk proteins to promote cell growth, spreading infiltration	Hewitt et al. (2019)
Bone	Poly (lactic acid) scaffolds with square pores	Meng et al. (2021)
	PCL MEW with square pores and chaotic gelatin SES scaffold	Wang et al. (2021)
	Calcium phosphate coated PCL scaffolds with square pores and fiber offset	Abbasi et al. (2020)
Cartilage	PCL structures with square pores for cell laden hydrogel reinforcement	Peiffer et al. (2020)
	PCL structures with square pores in combination with cytokine loaded PLGA microspheres	Han et al. (2020)
	Reinforced hyaluronic acid scaffold MEW PCL structure with square pores	Galarraga et al. (2021)
Blood vessel	Tubular PCL scaffold with square pores with aortic root features	Saidy et al. (2020)
	PCL electrospinning (chaotic) and MEW (rectangular pores) bilayered scaffold	Jungst et al. (2019)
Periodontal Tissue	PCL scaffolds with square pores with fluorinated calcium phosphate caoting	Daghrery et al. (2021)
Nerve tissue	Gold coated PCL scaffold with square pores	Wang Y et al. (2020)
	PCL scaffold with square pores and different surface modifications	Chen T et al. (2021)

Distinctive aspects, however, have to be considered to achieve the desired structural features of scaffolds in MEW approaches. Varying MEW scaffold architectures and their printing parameters are shown in Table 2.

**TABLE 2:** MEW structural design overview: Different scaffold architectures fabricated with MEW. Indicated are die dimension of building blocks, the fiber diameter and the processing temperature (in°C), the voltage (in kV), the distance between collector and nozzle (in mm), the printing speed (in mm s^−1^) and the material flow (in ml min^−1^ or bar).

MEW architecture	Dimension	Fiber diameter	Printing parameters						Citation
			Temperature	Pressure	Speed	Collect or distance	Voltage		
Box-Structure 90°	Box diameter: 100 µm	820 nm	84–109°C	2.8 bar	91.6 mm s^−1^	1.5 mm	2.9 kV		Hochleitner et al. (2015)
Serpentine Scaffold	Arc diameter: 0.5–1 mm	20 µm	75–85°C	2 bar	4.7 mm s^−1^	4 mm	6–6.5 kV		Saidy et al. (2019)
Box-Structure 10–45°	Fiber Spacing	10 µm	90°C	0.5 bar	11.5 mm s^−1^	20 mm	12 kV		Eichholz and Hoey, (2018)
	300 µm										
Offset-Box-Structure 90°	Box diameter: 250–750 µm	6–10 µm	80°C	20 ml h^−1^	-	10 mm	5–7 kV		Abbasi et al. (2019)
Hexagonal	Side length: 400–800 µm	20 µm	85–90°C	1 bar	4.5 mm s^−1^	3.5 mm	4.5 kV		Castilho et al. (2018)
Sinusoidal	Wavelength: 336 µm	27 µm	90–105°C	4 bar	2.2–3 mm s^−1^	4.5 mm	5.5 kV		Hochleitner et al. (2018a)
	Peak-to-peak: 139 µm										

To generate precise patterns, printing speeds close to or slightly above the CTS are used. Saidy et al. fabricated serpentine scaffold geometries by using a collector speed slightly above the CTS with a close to straight fiber flight path. The close to straight fiber path allows an exact control over the deposited fiber (Saidy et al., 2019). However, the mechanical stretching effect is lost when printing at CTS. Printing at CTS is only advisable, when continuous tool head motion is possible. When abrupt movement changes are needed, the printing speed needs to be adjusted. Castilho et al. fabricated scaffolds with hexagonal pores by varying the tool head speed. To reduce printing defects such as inconsistent material deposition with fiber coiling and looping fibers at the angled segments, a customized velocity profile was used. When approaching angled parts, the process was slowed down to CTS and sped up to two times CTS when printing straight parts. By modifying the speed in dependency of the desired structure, print defects can be avoided and fiber deposition precision can be increased (Castilho et al., 2018).

While most of the time printing speeds at CTS or above are used to print scaffolds, fiber coiling characteristics at speeds below the CTS can be advantageous to achieve certain mechanical properties. Hochleitner et al. used this strategy to fabricate sinusoidal patterns to mimic the biomechanical behavior of tendon in ligament. By varying the speed in a range of 65–100% of the CTS, sinusoidal to linear fibers with wavelengths in the range of ten to multiple hundred micrometers could be fabricated in a controlled manner, mimicking size and scale of native collagen fibers (Hochleitner et al., 2018a).

**FIGURE 2:**
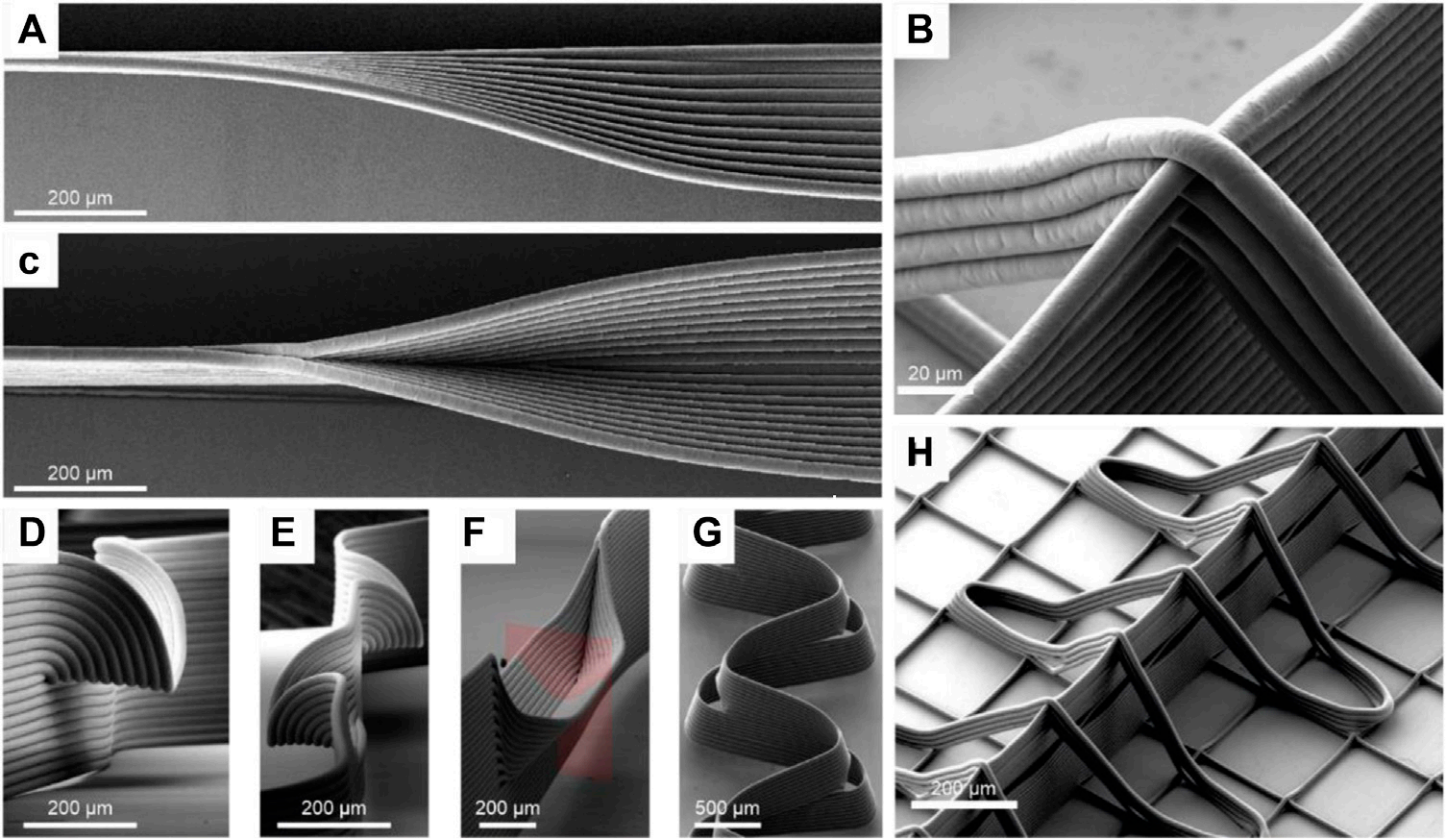
Microscale layershifting (Liashenko et al., 2020a) and out-of-plane-fiber-deposition (Ruijter et al., 2018): MEW structures such as overhangs **(A,D,E)**, branching walls **(C,F)**, tilted walls **(G)** are shown that are fabricated by deliberately offsetting the print path for each layer in a process termed “microscale layer shifting”. Images **(B,H)** show out-of-plane-fibers, fabricated to adapt material properties, enabled by the contactless fabrication characteristics of MEW.

Disadvantages of MEW resulting from the indirect fiber deposition due to the jet lag at printing speeds above CTS were on the other hand utilized to fabricate specialized patterns. Liasenko et al. used a special technique termed “microscale layer shifting” to print structures such as overhangs, branching and wall texturing and further they accomplished tilting of MEW fiber walls without support structures (Figure 2A,C–G). By deliberately offsetting the print path for each layer, fiber deposition can be manipulated in such a way that fibers are layered in a nonvertical manner (Liashenko et al., 2020a). Such advanced patterns can be important to adapt scaffold features to specific biomechanical or surface properties.

To minimize the deviation between the printed and programmed pattern caused by a jet lag during the printing process, Hrynevich et al. developed a geometrical model to predict the fiber deposition depending on the collector speed, the critical translation speed of the process and the flight path of the fiber. The model enables an up to ten times more accurate evaluation of the printing result in comparison with the programmed tool path. However, the model is limited to the first layer of a printing process and only to simple shapes. Sinusoidal structures could not be predicted with higher precision compared to the tool path (Hrynevich et al., 2020).

The fundamental fabrication principle of MEW as a contactless AM technique also allows the out-of-plane fiber deposition (Figure 2B,H). Ruijter et al. produced out-of-plane fibers that stabilized MEW walls to adapt the intrinsic shear modulus of MEW-hydrogel constructs. However, it should be noted that out-of-plane printing affects the fibers in different ways, depending on whether they were produced ascending or descending. The ascending fibers showed smaller span compared to the descending in dependency of wall height. (Ruijter et al., 2018).

**FIGURE 3:**
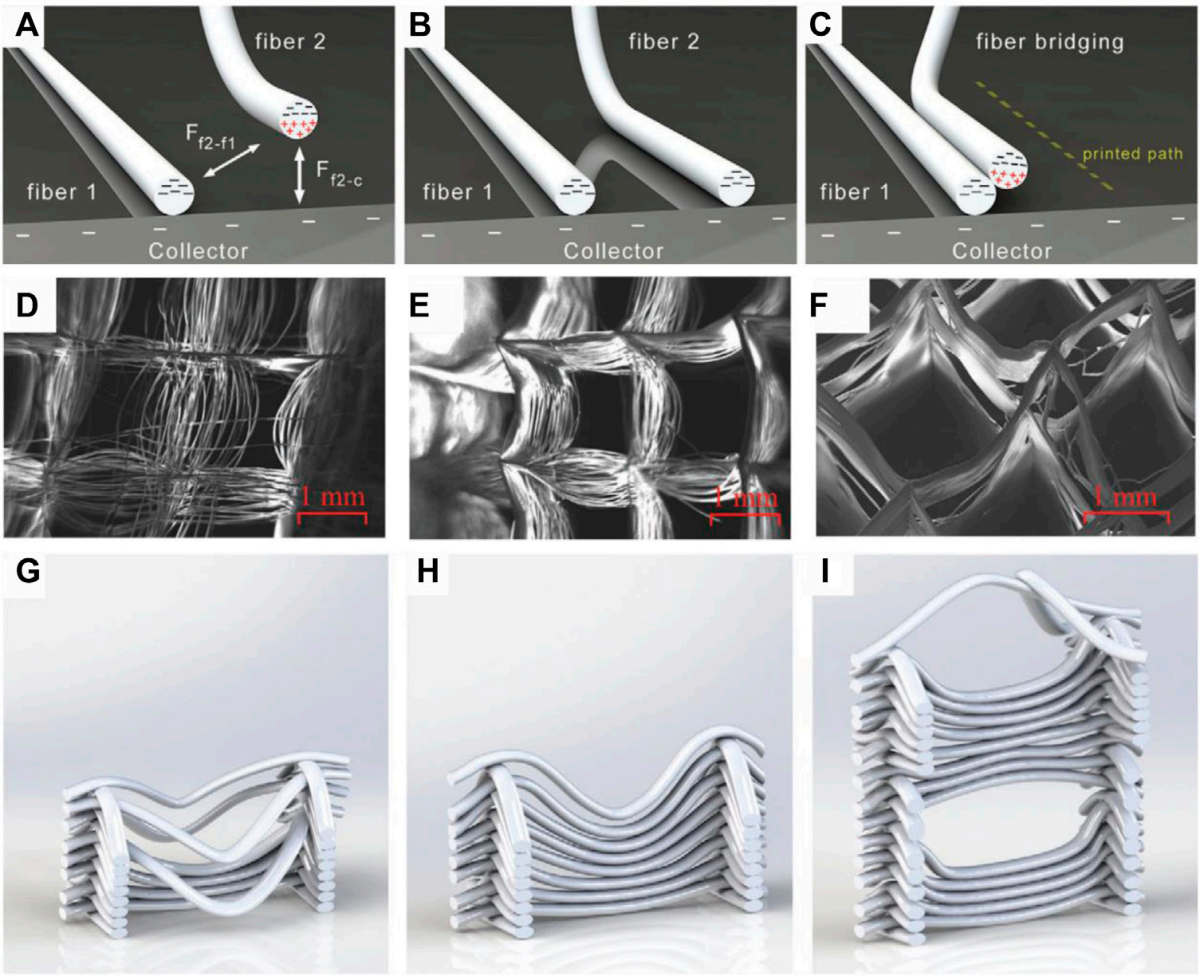
Fiber displacement caused by fiber attraction **(A–C)** (Kim et al., 2021) and fiber repulsion caused by collector temperature **(D–I)** (Nguyen et al., 2019):While airborne, electrostatic attractive forces are exerted from already deposited fibers as well as from the collector on newly formed fibers **(A)**. When the attracting effect of the collector dominates or inter fiber distance is large enough, the correct fiber deposition occurs **(B)**. If the inter fiber distance is small enough, the attracting forces of already deposited fibers are large enough to affect the fiber deposition of the newly formed fiber **(C)**. Fiber repulsion in dependency of the collector temperature with a low temperature (16°C) **(D,G)**, room temperature (23.5°C) **(E,H)** and a high collector temperature (30°C) **(F,I)**.

For the fabrication of highly porous scaffolds with a small fiber distance, intrinsic process limitations have to be considered. Due to the electrostatic forces between deposited fibers, a minimum inter-fiber distance must be ensured to prevent printing defects. Kim et al. and Ding et al. characterized the minimal fiber distance in dependency of the fiber diameter, layer count and conductivity of the print substrate. Due to the electrostatic forces towards already deposited fibers, newly deposited fibers are attracted towards neighboring fibers if the inter-fiber distance gets too small while using a conductive substrate. Residual positive charge of the fibers is transported quickly away and the deposited fibers themselves attract oncoming fibers (Figures 3A–C). This causes a smaller inter-fiber distance or fiber fusing, when new fibers are printed and when the working distance is set too small. On the other hand, repulsion of newly deposited fibers is observed due to residual charge of already deposited fibers, when using a non-conductive substrate. Remaining positive charge in the fibers affects deposition of new fibers and widens the inter-fiber distance. Attracting and repulsing forces correlate with the fiber diameter, with bigger fibers permitting only a larger inter-fiber distance before attraction or repulsion set in. Electrostatics also scales with the amount of layers, with increasing layer count permitting only a larger inter-fiber distance. (Ding et al., 2019; Kim et al., 2021). Cao et al. investigated the impact of the collector temperature to effect the polarization of deposited fibers to optimize accurate deposition of newly formed fibers. When printing multiple layers (>50 layers) at a low (16°C) or high (30°C) temperature, two forms of fiber disorder are observed depending on the printing parameters. When the collector temperature is low, incoming fibers are displaced laterally due to polarization of the electric field. In contrast, a high collector temperature leads to vertical repulsion (Figures 3D–I). (Cao et al., 2021)

To reduce fiber sagging, Nguyen et al. investigated the characteristics of deposited fibers in dependency of the collector distance, reservoir temperature and distance of orthogonal fibers. Higher temperatures of the polymer resulted in increased sagging and larger sagging angles while an increase in distance to the collector, caused by printing in multiple layers, reduced the sagging of fibers. The distance between orthogonal printed strands, reduced the fiber sagging with decreasing distance (Nguyen et al., 2019).

## Combination of MEW with other techniques

### Hydrogel printing

Hydrogels are an important vehicle for cell printing and mimic the environment of cultivated cells to native conditions. The adjustment of mechanical properties of hydrogels is essential to generate form stable printing products while maintaining cell growth, -motility, -differentiation and tissue formation (Lee and Yeong, 2016; Saroia et al., 2018). MEW offers the possibility to modify physical characteristics of multi-material constructs comprising fibers as well as hydrogels to enhance cell survival and ingrowth properties. Such multi-material MEW processes can be adapted to a wide range of mechanical properties, and thus enable target tissue specific adjustments to obtain ideal scaffold properties.

Reinforcing MEW frames embedded in hydrogel have an enormous effect on the toughness and elastic modulus of the construct, while only occupying a fraction of the volume. Bas et al. prepared hydrogel MEW constructs and achieved a hundredfold increase in elastic modulus and work of extension compared to scaffold-free hydrogels alone (Bas et al., 2017). The hydrogel requirements regarding the stiffness in such composite constructs are less demanding thus enabling a lower crosslinking, which in turn benefits the cell motility, proliferation and tissue formation. In comparison, a higher crosslinking density frequently causes limited cell mobility and therefore migration, but can also restrict proliferation and differentiation due to reduced porosity and diffusivity (Momot et al., 2017; Chimene et al., 2020; Ramiah et al., 2020).

As noted previously, supporting MEW walls and out-of-plane fibers (Figure 2B,H) can be utilized to adapt the shear modulus of hydrogel MEW constructs. By adjusting the wall properties, such as the amount of support fibers, the shear modulus in the hydrogel MEW construct can be multiplied compared to scaffold-free hydrogel alone (Ruijter et al., 2018).

Rujiter et al. investigated the convergence of MEW and extrusion-based hydrogel printing into a single manufacturing approach, allowing the simultaneous fabrication of MEW fibers and application with cell-encapsulated hydrogels. Precise x- and *y*-axis control of cell deposition was achieved as well as *z*-axis control of multiple layers. In an exemplary approach for cartilage tissue, the voltage necessary to perform MEW (5–15 KV) showed no effect on viability, metabolic activity or cartilage-like matrix production of the investigated differentiating equine-derived mesenchymal stromal cells (Ruijter et al., 2019).

### Combination of MEW with fused deposition modelling

FDM can also be used to enhance mechanical scaffold properties of MEW-generated scaffolds. The combination of both printing techniques can yield MEW-generated microscale fibers (3–20 µm) for cell adhesion and ingrowth as well as FDM-produced thicker fibers for mechanical support (200–600 µm). Such a combined-printed scaffold can be even generated in the same process without the need to change the nozzle, but requires a distinct printing protocol with switches between MEW- and FDM-specific printing parameters and thus has to vary the collector distance and material flow through the nozzle and provide an on and off switch of the electric field (Gao et al., 2020).

The combination of MEW and FDM also enables the tailoring of the scaffold properties such as the introduction of auxetic properties. Auxetic compounds have a negative Poisson ratio and expand in volume when stretched. This characteristic lets them undergo larger deformations and exhibit flexible properties under dynamic load. Jin et al. fabricated tunable auxetic scaffolds with traditional fused deposition modelling and combined them with ME-written fibers for increased cell adhesion and growth. Mechanical properties could be adapted by controlling the size and angle of the scaffold cell units (Jin et al., 2021).

MEW and FDM fibers can also be used to create channels in hydrogel structures to enhance nutrient, growth factor or therapeutics supply. Haigh et al. and Wand et al. used MEW and FDM generated PCL fibers submerged as a sacrificial material in hydrogels. After crosslinking of the hydrogel, the PCL fibers were dissolved with acetone and water mixture thus structuring the gel with microchannels (Haigh et al., 2016; Wang S et al., 2020).

### Solution electrospinning

In addition, MEW can be combined with SES for specific applications. While SES can generate randomly oriented nanofibers with dense fiber networks, MEW can be utilized to guarantee fiber orientation and deposition. Jungst et al. fabricated a bilayered tubular scaffold consisting of an inner electrospun dense fiber mesh and an outer lay of melt electro spun microfibers with controlled deposition and orientation. This combination of techniques allows fiber diameter varying by one order of magnitude within the same scaffold. By using different pore geometries and fiber diameter within the same scaffolds, specific cell morphologies and differentiation of endothelial cells and vascular smooth muscle cells were supported in the respective layers (Jungst et al., 2019).

### Adaptions of MEW to produce specifically molded scaffolds

To achieve adequate scaffolds via MEW, printing on a flat collector is often insufficient. Therefore, it is necessary to adapt the collector to the specific scaffolds characteristics that are needed for clinically relevant applications.

Custom-made printing substrates and molds are necessary for the fabrication of scaffolds that can mimic the anatomical model. For precise fabrication of curved contour meshes on a nonplanar collector, Saha et al. characterized the influence of nozzle distance and strength of the electrical field for curved surfaces. He placed a non-conductive polylactid acid dome on a flat conductive collector to investigate fiber deposition properties and could show, that an accurate fiber deposition is achievable by maintaining a constant electrical field through a constant vertical distance between nozzle and collector (Saha et al., 2021).

Peiffner et al. investigated the impact of conductive and non-conductive molds for clinically relevant medial human femoral condyle implants. Printing onto non-conductive molds led to significant smaller fiber diameters with a cylindrical cross section. Fibers printed on conductive molds turned out to be bigger and had an ellipsoidal cross section due to a slightly higher electrical field strength caused by the conductive properties of the mold. An impact of the mold thickness on the fiber thickness and deposition accuracy was not observed (Peiffer et al., 2020).

**FIGURE 4:**
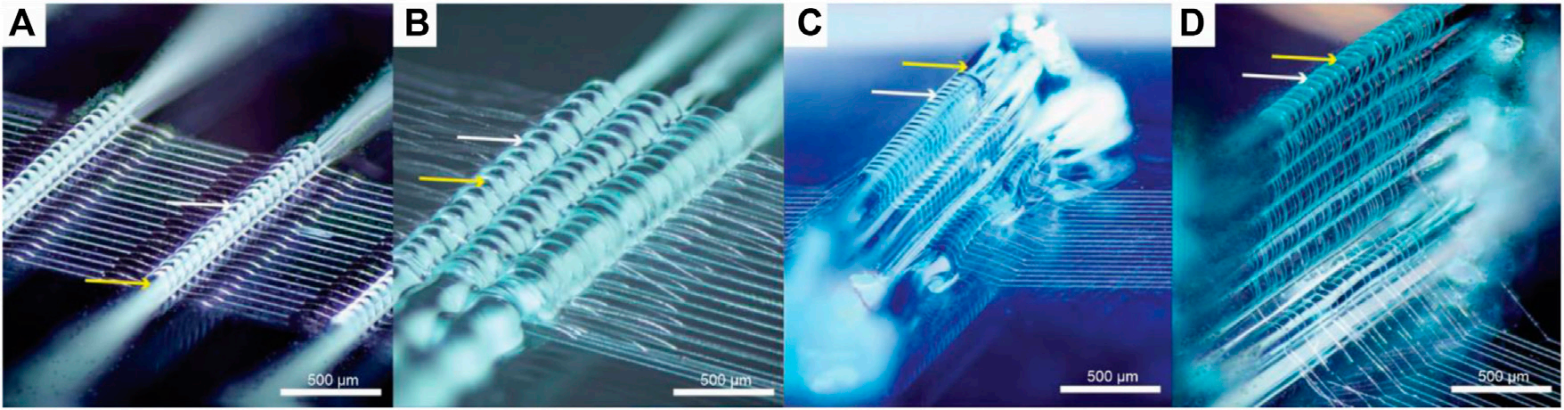
Melt electro writing on top of hydrogel strands (Pluronics F127) (Ruijter et al., 2019). Deposited MEW fibers on a single strand **(A)**, grouped strands **(B)** and multiple layers of Pluronics F127 **(C,D)**. White arrows indicate the melt electro spun PCL fibers and yellow arrows depict Pluronics F127 strands.

Another method to print on an adaptable print bed conformation was carried out by Ruijeter el al. Pluronics F127 was used to guide the fiber deposition of MEW fibers on a 3D support structure to enable printing on a non-planar surface (Figure 4). While precise control over the spatial deposition of fibers remains challenging, fibers were successfully deposited on top of multiple layers of hydrogel (Ruijter et al., 2019). The use of Pluronics also allows their use as sacrificial material due to their unique solution-gel phase transition (Suntornnond et al., 2017). If the temperature falls below the critical gelation temperature (CGT), the hydrogel liquefies and can be separated from the scaffold and the desired MEW structure is maintained.

When using complex 3D objects with a higher impact on the electrical field, extensive adaptions to the MEW process are needed. O’Connell et al. characterized the fiber deflection in the MEW process near hemispheres out of different materials. He established a tool-path algorithm to modulate the nozzle path to pre-emptively correct the electrostatic deflection caused by these hemispheres. The fiber deflection in such printing conditions is usually material-independent but it is influenced by the distance to the object and the surface area of the object and the collector distance. Materials only then impact this process, when their charge relaxation times are shorter than the printing times (O'Connell et al., 2021).

**FIGURE 5:**
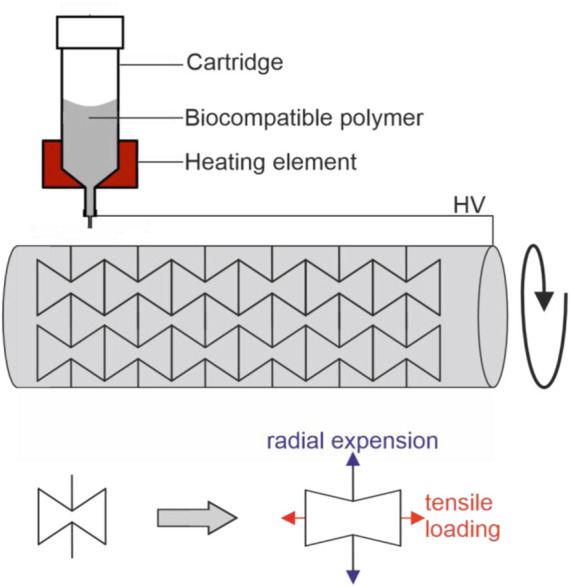
Fabrication of auxetic tubular microfibre scaffolds, fabricated via melt electrowriting, adopted from (Paxton et al., 2020): By using a tubular print bed, one of the axes of a conventional 3D printer is exchanged for rotating mandrel. In such a setup, the rotation speed has to be consistently adjusted with increasing numbers of layers to maintain stable printing conditions. Printing of auxetic structures lead to scaffold structure that increases in diameter (indicated in blue) when stretched (indicated in red).

Most MEW studies are carried out on a flat collector with straight fiber deposition (Hochleitner et al., 2015; Eichholz and Hoey, 2018; Hrynevich et al., 2018; Abbasi et al., 2019; Xie et al., 2019; Jin et al., 2020). In contrast, tubular shaped print beds are needed for the fabrication of scaffolds with an anatomically relevant shape suitable for tubular shaped scaffolds such as vessels, heart valves or aortic roots. Here a rotating mandrel can be used as a collector instead of a flat surface but this demands different fiber deposition properties in comparison to a flat collector. For example, printing parameters such as rotation speed have to be constantly adjusted to maintain stable printing conditions, because the effective collector speed increases with increasing distance to the collector. Therefore, the printing speed has to be adapted in every layer to achieve stable printing conditions. Mieszczanek et al. fabricated tubular structures of 12 mm diameter consisting of 300 layers by maintaining a specific winding angle and using a constant electrical field (Mieszczanek et al., 2021). A tool to generate G-code movement for tubular collectors and tool heads was introduced by McColl et al. to guarantee matching layer-by-layer deposition (http://mewtubes.herokuapp.com) (McColl et al., 2018). This is necessary, because the MEW process cannot be stopped and the scaffold architecture and every layer must be coordinated to enable well-regulated structures. The potential of a tubular collector also allows fabrication of micrometer scale tubular scaffolds with specific properties such as auxetic structures that increase in diameter when stretched (Figure 5). (Paxton et al., 2020)

**FIGURE 6:**
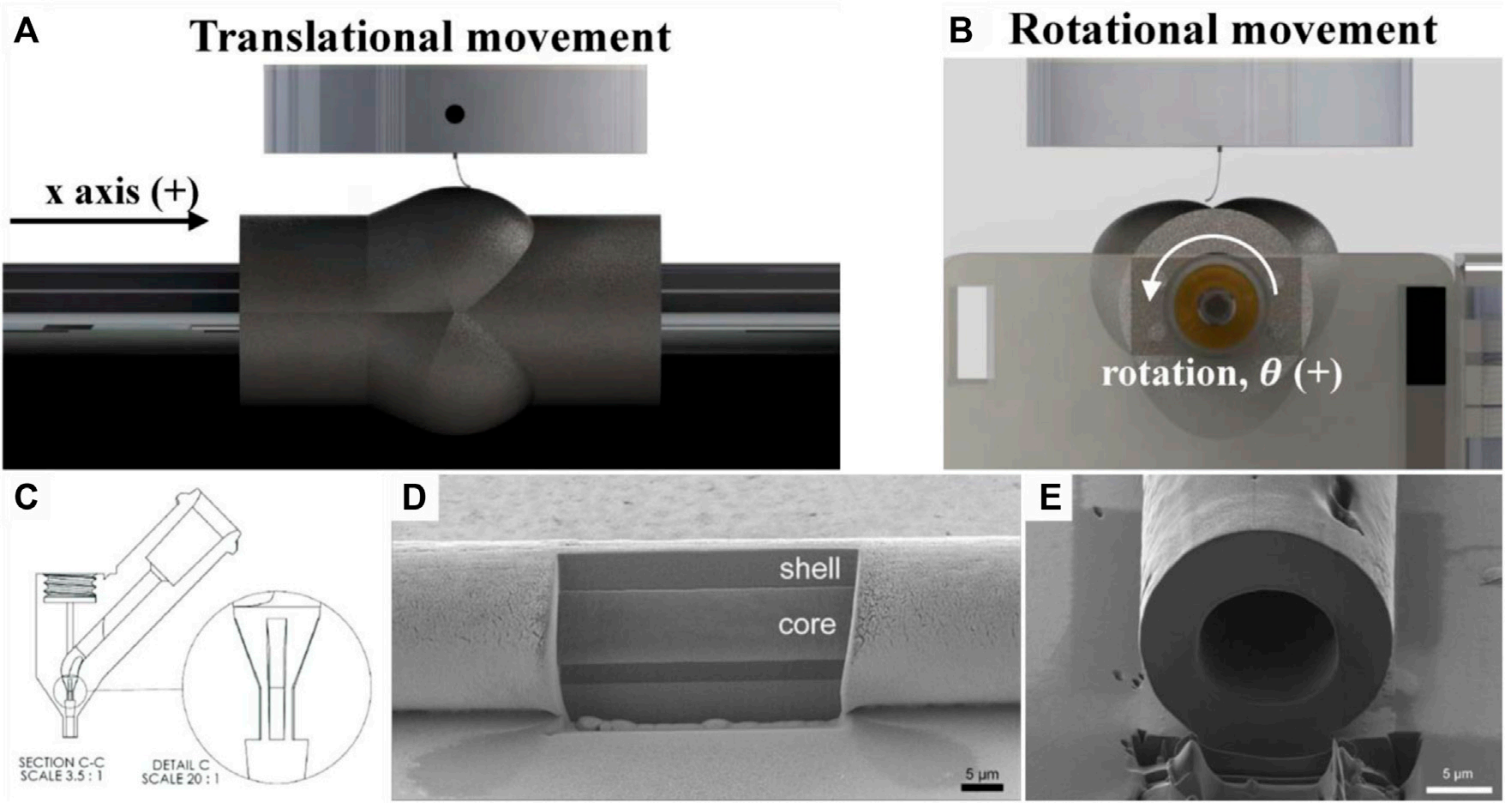
Cylindrical MEW printing bed with aortic sinus features **(A,Β)** (Saidy et al., 2020), coaxial nozzle **(C)** for hollow MEW fiber fabrication **(D,E)** (Eberle et al., 2021)

Saidy et al. used a two-component tubular collector consisting of an electrically conducting aluminum core and a non-conducting polylactic acid shell with features of aortic sinuses to print patient specific aortic root scaffolds (Figure 6A,B). For this application, especially the usage of a non-conductive shell with specific tissue features enabled the establishment of a stable electric field while using a non-rotationally symmetrical collector, whereas the usage of a fully conductive collector often led to fiber instabilities such as pulsing and long beading due to variations of the electrical field (Saidy et al., 2020).

### Nozzle modifications in MEW

The nozzle form and size can be adapted in MEW to achieve either hollow fibers or to effect physiochemical fiber properties such as specific surface modifications useful for drug delivery systems or other specific mechanical properties of the fibers. Eberle et al. printed hollow PCL fibers with an outer diameter of 10 µm and an inner centered hollow channel of 6 µm with a custom designed selective laser melting (SLM) printed coaxial nozzle (Figures 6C–E). One could imagine that MEW with a coaxial needle could be combined with printing on a rotating printing bed to obtain hollow fibers on a tubular printing bed. Nevertheless, printing hollow fibers with MEW requires a very low printing pressure with highly precise pressure control to achieve stable printing conditions without rupturing the Taylor cone (Eberle et al., 2021).

The nozzle-exit shape can also be modified to affect fiber diameter of deposited fibers. Esmaeilirad et al. used different nozzle-exit-channel shapes to examine the impact on the generated fiber diameter. Among the nozzle-exit-channel investigated, the rectangular nozzles were able to generate the smallest fibers with a reduction in fiber diameter of up to 50% compared to circular opening with comparable nozzle-exit-channel cross section area (Esmaeilirad et al., 2017).

Wunner et al. characterized the influence of gravity to MEW and showed that MEW is reproducible from all directions. While printing sideways, top or upside, down configuration does have an impact on the precise fiber deposition, the effect can be controlled by adjusting printing parameters (Wunner et al., 2018a).

## Materials in MEW

While many thermoplastic materials are suitable for fabrication with MEW, Polycaprolacton (PCL) is the mostly used material in MEW, due to its biodegradable, semicrystaline properties with a low melting temperature (Janmohammadi and Nourbakhsh, 2019). Nevertheless, other materials are developed and used in MEW for scaffold fabrication. An overview over the different materials that are suitable for MEW is shown in Table 3.

**TABLE 3:** Materials used in MEW: Different materials used in MEW for Scaffold fabrication or medical implants. Indicated are the processing temperature (in°C), the voltage (in kV), the distance between collector and nozzle (in mm), the printing speed (in mm s^−1^) and the material flow (in ml min^−1^ or bar).

Material	Fiber diameter	Processing parameters	Young´s modulus	Maximum strength	Elongation at break	Citation
Poly (hydroxymethylglycolide-co-ε-caprolactone (pHMGCL) in combination with PCL	4–7 μm	87°C, 7 kV 3–5 mm, 25 mm s^−1^, 1–4 bar	-	-	-	Castilho et al. (2017)
Poly (l-lactide-co-ε-caprolactone-co-acryloyl carbonate) (poly (LLA-ε-CL-AC)	25 µm	145°C, 7.0 kV, 4.5 mm, 7 mm s^−1^, 3.0 bar	314 MPa	53 MPa	90%	Chen et al. (2016)
Poly (lactic acid) (PLLA)	40 µm	190°C, Collector: 0.5 kV, Needle: 1.5–4 kV, 3 mm, 0–45 mm s^−1^, 0.2–2 bar	1,672.2 MPa	66.6 MPa	5%	Meng et al. (2021)
Poly (urea-siloxane) (PUS)	10–17 µm	80–100°C, 8–12 kV, 8.5 mm, 25–66 mm s^−1^, 0.05–0.25 ml h^−1^	27.3 MPa	2.04 MPa	773%	Hochleitner et al. (2018b)
Poly (l-lactide-co-ε-caprolactone) (PLCL)	14–40 μm	Preheating 150°C for 24–48 h	5 MPa	2–4 MPa	500%	Sanchez Diaz et al. (2021)
		110°C, 5.4–6 kV, 3–5 mm, 1.6–45 mm s^−1^, 0.3–0.9 bar				
Polybutylene succinate (PBS)	30 µm	235°C, 60 kV, 100 mm	300 MPa	25 MPa	14%	Ostheller et al. (2021)
		0.1 ml/min				
Poly (2-ethyl-2-oxazine) (PEtOzi)	45 µm	150°C, 2–4 kV, 3.3 mm, 21–30 mm s^−1^, 1.5–2 bar	0.14–0.2 MPa	-	-	Nahm et al. (2020)
Poly (2-ethyl-2-oxazoline) (PEtOx)	8–138 µm	200–220°C, 3.0–7.0 kV, 3.0–7.0 mm, 3.3–6.6 mm s^−1^, 1–3 bar	-	-	-	Hochleitner et al. (2014)

A blend with PCL to improve material properties was produced by Castilho et al. This group used poly (hydroxymethylglycolide-co-ε-caprolactone) (pHMGCL) with different purposes. This blend is more hydrophilic, presents a tunable degradation rate and offers potential for biofunctionalisation with regard to the later scaffolds (Boere et al., 2014; Boere et al., 2015). By these characteristics, pHMGCL scaffolds promote the cellular alignment of cardiac progenitor cells. While the blend afforded an adaptation of the printing parameters with regard to the melting temperature, the CTS and the necessary acceleration voltage, the fabricated fibers were in the range of typical PCL fibers (Castilho et al., 2017).

MEW of a PCL protein blend was carried out by Hewitt et al. Milk protein in the form of lactoferrin and whey protein were blended with medical grade PCL to fabricate scaffolds for deep tissue dermal regeneration. Cell growth, spreading and scaffold infiltration of human keratinocytes and dermal fibroblasts was thus significantly increased (Hewitt et al., 2019).

A photo-cross-linkable polymer was used by Chen et al. Poly (l-lactide-co-ε-caprolactone-co-acryloyl carbonate) (poly (LLA-ε-CL-AC)) is a photo-cross-linkable and biodegradable polymer with high preservation of modulus and stiffness under repetitive loading conditions after UV-crosslinking. The Young’s modulus of 370 MPa and the adaptable mechanical properties by variation of the copolymer content makes poly (LLA-ε-CL-AC) a promising scaffold material for soft connective tissue engineering applications in general (Chen et al., 2016).

Another thermoplastic material that is processible via MEW is poly (lactid acid) (PLLA). Meng et al. produced PLLA scaffolds with a fiber diameter of 40 µm and a pore size of 200 µm for application in bone tissue engineering. Due to thermal degradation of the molten PLLA during the MEW process, a reduction in molecular weight of about 10% and melt viscosity of 15% was observed. Nevertheless, scaffolds with osteoconductive properties that promote bone marrow cells differentiation were fabricated. Fabricated PLLA scaffolds exhibited a tensile strength of 66,6 MPa, a Young´s modulus of 1,672 MPa and an elongation at break of 5% (Meng et al., 2021).

A thermoplastic elastomer that can be processed via MEW was used by Hochleitner et al. Poly (urea-siloxane) is a (AB)n-type thermoplastic elastomer consisting of soft poly (dimethylsiloxane) segments and hard urea units. A comparison with the material properties showed a Young’s modulus of 2 MPa, corresponding to one 10th of the Young´s modulus of PCL, and a strain of 2 MPa and elongation at break of 770% compared to the initial length. The resulting fiber diameter of Poly (urea-siloxane) during printing is also dependent on the applied voltage, contrary to other polymers. With this material, no fiber sagging was observed leading to constructs with a homogenous build height (Hochleitner et al., 2018b).

Another processible elastic polymer is Poly (l-lactide-co-ε-caprolactone) (PLCL). PLCL is an elastic, heat sensitive polymer with high melt viscosity and tunable degradation properties. Sanchez Diaz et al. used a thermal pre-treatment to induce thermal degradation and tailor the melt viscosity to make PLCL utilizable in MEW. Depending on the thermal pretreatment, a tensile strength of 2–6 MPa, a Young´s modulus of 5–7 MPa and an elongation at break of 200–500% was measured for ME-written PLCL scaffolds. Especially tissue with mechanically dynamic environments such as skin or cardiac and lung tissue could benefit from the elastic properties of PCLC (Sanchez Diaz et al., 2021).

A hydrogel that can be processed by MEW is poly (2-ethyl-2-oxazine) (PEtOzi). Nahm et al. characterized the processing of PEtOzi via MEW and its properties after the printing process. The crosslinking of PEtOzi is based on a thermally reversible Diels–Alder click chemistry and after cooling the equilibrium is shifted to the bicyclic adduct with high shape persistence. Upon hydration, the printed hydrogel swells to a volume of 290% compared to the dried state with a water content of 74%. Scaffolds produced with PEtOzi are remarkably robust due to chemical crosslinking and fiber fusion and exhibit a Young’s modulus of 0.14–0.2 MPa, comparable to hydrogels with similar water content (Nahm et al., 2020).

## Conclusion

MEW is a promising technique for fabrication of micrometer scale features of scaffolds and for expansion of 3D printing prospects for TE applications. MEW is based on the electrostatic attraction of micrometer fibers to a corresponding collector. Since MEW is an intrinsic multi-parametric process whose printing characteristics depend on the interplay of many factors, it is possible to adjust accurately the fiber deposition, the fiber diameter and the layer-by-layer deposition by adapting printing parameters. These parameters are printing speed, collector distance, voltage, the temperature of the polymer and of the print bed, adjusted to the specific desired structure. Further improvement in fiber diameter reduction and fiber deposition accuracy can be achieved through adaptions of solution and melt electrospinning strategies, which opens the possibility to mimic ECM fibers known from physiological connective tissue. Such strategies are nozzle modifications that allow a more precise and tunable material flow (Großhaus et al., 2020), deflecting electrodes that control the fiber deposition of airborne fibers (Liashenko et al., 2020b) and polymer additives that affect viscosity and conductivity. Using all these measures, material flow rate and collector attraction properties (Malakhov et al., 2015; Koenig et al., 2020) as crucial printing properties can be improved. MEW thus enables precise fabrication of tissue specific scaffolds. Shortcomings of MEW include a restricted material selection but with a steadily increasing number of newly developed materials for specific application, some of them with the potential for specific biofunctionalisation for sucessful cell seeding. Altered MEW printing strategies such as combination with FDM, using molds as printing bed, using a rotating printing bed are further interesting approaches to create complex geometries with high similarity to anatomic structures. Altogether, MEW opens the possibility to generate predictably scaffolds for high-resolution application specific tissue engineered products.
